# The use of computer‐aided design and manufacture for foot orthoses: A cross‐sectional study of orthotic services in the UK

**DOI:** 10.1002/jfa2.70031

**Published:** 2025-02-05

**Authors:** Laura Barr, Jim Richards, Graham J. Chapman

**Affiliations:** ^1^ Orthotic Department NHS Greater Glasgow and Clyde Gartnavel General Hospital Glasgow Scotland; ^2^ Allied Health Research Unit University of Central Lancashire Preston UK

**Keywords:** CAD/CAM, foot orthoses, insoles, orthotics

## Abstract

**Objective:**

This study aimed to identify how computer aided design and manufacture (CAD/CAM) technologies are currently being used for insole production by UK orthotic services in the National Health Service (NHS), including any variation in the specific processes and identify barriers to implementation.

**Design:**

A cross‐sectional study was undertaken using freedom of information requests sent to all 214 NHS Trusts and Health Boards (HBs) across the UK. The request comprised 22 questions relating to CAD/CAM for insole production by UK NHS orthotic services during the 2021/22 financial year.

**Outcome Measures:**

Analysis was undertaken and presented in terms of response rate to individual questions. Where free text responses were provided, thematic analysis was conducted.

**Results:**

Responses were received from 186 (86.9%) Trusts/HBs, those who did not have an orthotic service were excluded, and 131 responses were included in the final analysis. 70.5% (91/129) of Trusts/HBs used CAD/CAM to manufacture bespoke insoles. The most common workflow associated with CAD/CAM insole production was foot‐shape capture with a foam box impression cast (86.8% (79/91)); casts transported to another site (90.8% (79/87)); foam boxes scanned into a CAD/CAM system (81.6% (71/87)); insoles designed by a technician (73.6% (67/91)) and insole produced with reduction milling (59.1% (SD 37.92)). The greatest barriers to the use of CAD/CAM were those of equipment costs and staff experience and training.

**Conclusions:**

UK orthotic services have widely adopted CAD/CAM insole production, but fully‐digital workflow is uncommon. Hybrid‐digital workflow involves physical casts and their transportation, generating waste and impacting sustainability. Further research is required to understand how hybrid‐digital and fully‐digital workflow affect patient treatment outcomes, costs and sustainability. Barriers to CAD/CAM including costs and staff training which should be considered alongside the growing body of research around CAD/CAM technologies.

## 1 INTRODUCTION

Foot orthoses, known commonly as insoles, are used to treat many conditions of the foot and lower limb arising from various pathologies including musculoskeletal conditions, diabetic foot disease and traumatic injury [1, 2, 3]. Historically, bespoke insoles have been manufactured using traditional methods which require a physical cast of the foot using single use materials such as phenolic foam and plaster of Paris over which the final insole is then molded, this process can be time consuming, messy and produces waste products [4]. Since the 1980's the use of computer aided design and manufacture (CAD/CAM) has replaced certain elements of traditional manufacture in the prosthetic and orthotic industry, with an increasing trend towards the use of CAD/CAM throughout the medical industry in more recent years [5, 6]. The use of CAD/CAM in orthotic manufacture has long since conceptualised advantages with regard to improved accuracy of body shape capture, repeatability, improved quality and faster production times [4, 7, 8]. In addition to the initial advantages foreseen with the use of CAD/CAM, the Covid‐19 pandemic also instigated a change in perception around the benefits of this technology for reduced patient contact time during the assessment process [7, 9], and in conjunction with other digital technologies, offered the ability to provide a fully virtual service for patients requiring duplicate or repeat prescription of their orthoses [4, 7]. Beyond the pandemic, these benefits can be appreciated in terms of reducing unnecessary patient travel for face‐to‐face hospital visits in the long‐term [4].

Despite the purported benefits, barriers to CAD/CAM have been raised in the literature with regard to equipment costs, requirement for clinical training, and adaptation of orthotic workflow [10], fueled by a self‐reported lack of CAD/CAM expertise in the orthotic workforce [11]. Doubt has also been cast on the change in clinical processes instigated by the perceived lack of clinical experience with CAD/CAM technology, resulting in the insole design often being undertaken by a technician at a central fabrication site rather than the orthotist at the point of patient contact [7]. To the authors' knowledge, it is still not understood how widely CAD/CAM is being used in the UK for the production of bespoke foot orthoses, and how the UK workforce has adapted to incorporate such digital workflows, specifically with regard to the individual processes used within the CAD/CAM supply chain. Questionnaires regarding CAD/CAM in the orthotic industry have typically focussed on both prosthetic and orthotic services without a specific focus on foot orthoses, and have not had a high response rate from the UK orthotic workforce [4, 12].

This study aims to improve our understanding of how CAD/CAM technologies are currently being used for insole production by UK National Health Service (NHS) orthotic services. We aimed to identify any variation in the specific processes associated with a CAD/CAM workflow, and any barriers for implementation, in order to determine where future research should be directed.

## 2 METHODS

A cross‐sectional study was undertaken using the UK freedom of information (FOI) act to gather data [13] and reported in accordance with the STROBE cross‐sectional reporting guidelines [14]. From November 11, 2022 to December 2, 2022 FOI requests were sent to all 214 NHS Trusts and Health Boards (HBs) across the UK. The request comprised 22 questions (see Supplementary File 1 in Supporting Information S1) designed to gather information relating to UK NHS orthotic services during the 2021/22 financial year from April 6, 2021 to April 5, 2022. Not all questions required an answer, and Trusts/HBs were instructed on which specific questions they should answer depending on their particular responses. The request focussed on two main areas (1): CAD/CAM insoles and (2) barriers/facilitators to using CAD/CAM.

(1) CAD/CAM insoles

The aim of this section was to gather information on the volume of bespoke insoles prescribed by the Trust/HB, the methods used for manufacture, and the proportion of insoles manufactured by traditional and CAD/CAM methods. Further questions then explored the workflow relating to manufacture of CAD/CAM insoles; this included questions on the methods used to acquire digital foot models, the transportation of foot models, the design, and the manufacture of the insoles.

(2) Barriers/facilitators to using CAD/CAM

The aim of this section was to understand the reasons why services chose to use or not to use CAD/CAM as part of their insole manufacture process. Using previous publications which examined barriers and facilitators for the use of any CAD/CAM systems in the prosthetics and orthotics industries [4, 12, 15], a list of options was compiled from which respondents could either choose their answers, or provide a free text comment. Given recent considerations to the use of digital technology in supporting health services following the Covid‐19 pandemic [4], we also chose to include options regarding any benefits that CAD/CAM insole systems provided to Trusts/HBs during and following the pandemic. Approval for the study was received from the Health Ethics Review Panel at the University of Central Lancashire (HEALTH 0365 Phase 2).

### 2.1 Data analysis

An analysis was undertaken and presented in terms of response rate for the individual questions. Where free text responses were provided, the answers were reviewed and an inductive approach was used to form a thematic analysis [16], the themes of which were agreed by the authors and presented alongside anonymised quotations. Where questions required a numerical answer, if a respondent provided a range of values then the mean of those values was used in the analysis. Where numerical answers were provided, distribution of those values was analyzed using Kolmogorov‐Smirnov tests and presented as median values when data was not normally distributed. Where Trusts/HBs were asked to select one preferred method of shape capture, analysis was made on the assumption that a minimum of 51% of their CAD/CAM insole production would be manufactured using this method, and where two options were selected the subsequent analysis was based on the assumption that 50% of their CAD/CAM insole production would be manufactured using each method.

### 2.2 Patient and public involvement

No patients or members of the public were involved in the design of this study. Before dissemination across the UK, the FOI request was piloted by orthotists in three Trusts/HBs who provided comments on the content and structure of the questions; all comments were addressed in the final version of the FOI request.

## 3 RESULTS

### 3.1 Response rate

**FIGURE 1:**
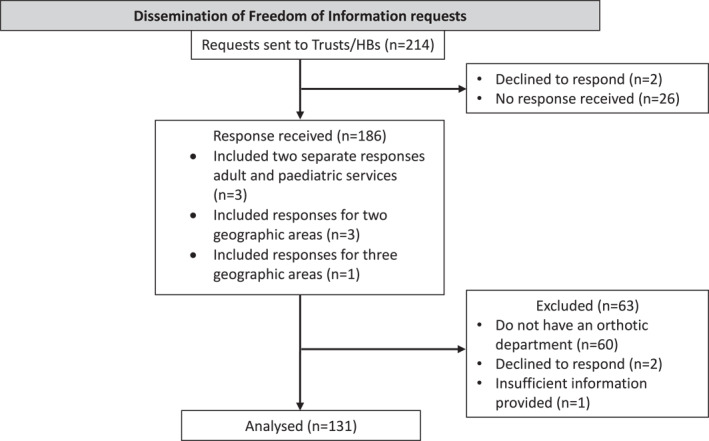
Study flow chart.

Complete or partially complete responses were received from 186 (86.9%) Trusts/HBs, two (0.9%) declined to respond, and 26 (12.2%) provided no response. On preliminary review of the responses, 60 stated that they did not have an Orthotic Department in their Trust/HB and were excluded from the analysis. Within the received responses one was excluded due to lack of information as only one question was answered despite prompting to complete further questions. Three Trusts/HBs provided separate responses for their adult and pediatric services, three provided individual responses for two separate geographical areas within their Trust/HB, and one provided individual responses for three geographical areas within their Trust/HB. Therefore, the total number of responses included in the analysis was 131 (Figure 1). The geographical regions of the respondents are presented in Table 1. Not all Trusts/HBs provided answers to all questions requested of them, with the variation in response rate documented in Supplementary File 2 in Supporting Information S2.

**TABLE 1:** Responses by geographical region.

Region	Number of respondents	Percentage of total (131)
Scotland	11	8.4%
Northern Ireland	4	3.1%
Wales	7	5.3%
England		
North East	7	5.3%
North West	14	10.7%
Yorkshire and Humber	11	8.4%
East Midlands	7	5.3%
West Midlands	10	7.6%
East	11	8.4%
London	17	13%
South East	19	14.5%
South West	13	9.9%

### 3.2 Overview of bespoke insole provision

Responses showed that a greater proportion of Trusts/HBs (61.8% (81/131)) provided a contracted orthotic service, whereby the NHS pays for orthotic services from an external company, with approximately 30% (31.3% (41/313)) of Trusts/HBs using an in‐house service where orthotists are employed directly by the NHS, and a small number (6.9% (9/131)) used a combined contracted and in‐house service (Figure 2a).

FIGURE 2(a–i) Proportionate answers to individual questions from Trusts and Health Boards (HBs). (a) Which of the following best describe your orthotic service? (b) Does your orthotic service provide bespoke insoles to patients? (c) Does your orthotic service ever use foam box impression casts to capture the shape of the patient's foot, when prescribing CAD/CAM insoles? (d) Is the negative foam box impression cast usually scanned into the CAD/CAM system, or is it filled with plaster first and then the positive model scanned? (e) Are the foam box impression casts usually transported to another site to be scanned into the CAD/CAM system? (f) Does your orthotic service ever use slipper casts/plaster casts to capture the shape of the patient's foot, when prescribing CAD/CAM insoles? (g) Are the slipper casts/plaster casts usually transported to another site to be filled with plaster and scanned into the CAD/CAM system? (h) In your orthotic service, which is the most common method used to capture the shape of the patient's foot, when prescribing CAD/CAM insoles? (i) Who is usually responsible for performing the modeling/rectification of the CAD/CAM insoles that your orthotic service provide?

**Figure:**
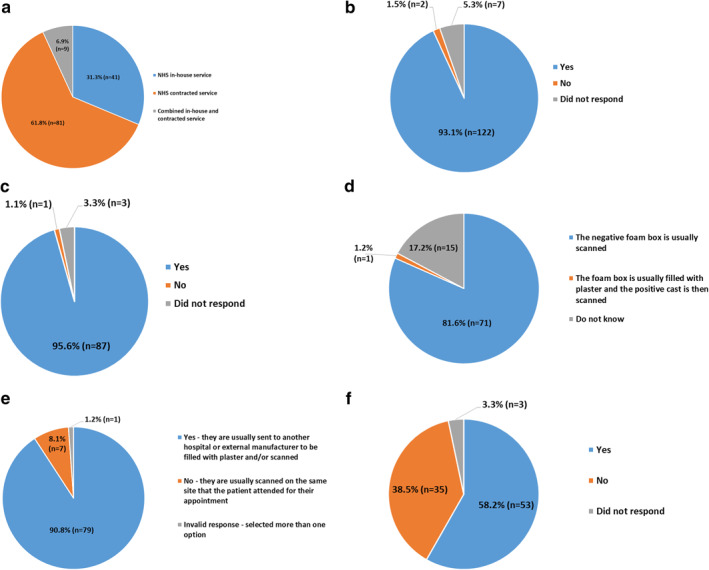

**Figure:**
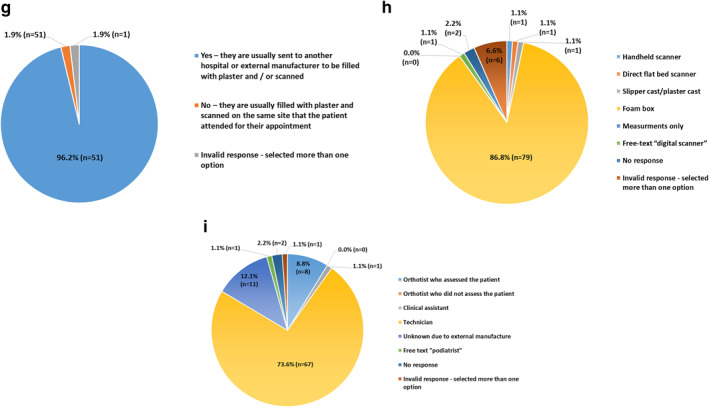

Of those Trusts/HBs that provided insoles, the majority (93.1% (122/131)) confirmed that they provided bespoke insoles to patients and a small number (5.3% (7/131)) did not respond (Figure 2b). Of those Trusts/HBs who did provide bespoke insoles, the majority (80.6% (104/129)) provided details of the number of bespoke insoles ordered for patients in the 2021/22 financial year. Fifteen of the 129 Trusts/HBs provided an estimated number or a range of values. The total number of bespoke insoles provided by Trusts/HBs was 144,414 (median 904.50, IQR 360.50–1652.25). Of those Trusts/HBs who provided bespoke insoles, 70.5% (91/129) used CAD/CAM whereas ∼20% (25/129) did not use CAD/CAM to manufacture the bespoke insoles and 10.1% (13/129) did not respond.

### 3.3 CAD/CAM insoles

Of the 91 Trusts/HBs that used CAD/CAM for insole manufacture, the response rate varied from 79.1% to 86.8% for the breakdown of the manufacturing methods used in their services. Six (6.6%) Trusts/HBs were unable to provide specific details due to the insoles being manufactured externally without the Trust/HB having knowledge of the external processes. A full breakdown of the manufacture methods are shown in Table 2.

**TABLE 2:** Techniques used to manufacture bespoke insoles.

Method of insole manufacture (respondents)	Volume of insoles: Median percentage^a^	Volume of insoles: Total	Volume of insoles: Total	Volume of insoles: Median total^a^
In‐house traditional (79/91)	0.0 (0.0–0.0)	11,006.89	27,296.65 (traditional manufacture)	0.00 (0.00–0.00)
Outsourced traditional (72/91)	3.0 (0.0–9.1)	16,289.76		11.71 (0.00–942.90)
In‐house computer aided manufacture using reduction manufacture (79/91)	0.0 (0.0–0.0)	22,044.63	76,381.25(CAD/CAM manufacture)	0.00 (0.00–0.00)
In‐house computer aided manufacture using additive manufacture (79/91)	0.0 (0.0–0.0)	5373.27		0.00 (0.00–0.00)
Outsourced computer aided manufacture using reduction manufacture (72/91)	78.0 (17.8–95.0)	44,320.25		400.00 (0.00–942.90)
Outsourced computer aided manufacture using additive manufacture (77/91)	0.00 (0.0–10.0)	4643.10		0.00 (0.00–49.61)

^a^ Median (IQR 25–75).

With regard to the number of years that CAD/CAM had been used as part of their insole manufacture process, 85.7% (78/91) Trusts/HBs reported a median of 10.00 years (IQR 7.5–15.00). The final set of questions in this section were designed to understand details of the CAD/CAM workflow. A high majority (95.6% (87/91)) of Trusts/HBs confirmed they sometimes used foam box impression casts when prescribing CAD/CAM insoles, and 3.3% (3/91) did not respond (Figure 2c). Of those who used foam box impression casts, 81.6% (71/87) scanned the cast directly into the CAD/CAM system, 1.4% (1/87) filled the cast with plaster before scanning, and 17.2% (15/87) did not know the specific processes due to this being undertaken by external manufacturers (Figure 2d). With regard to the location of scanning, 90.8% (79/87) reported that the foam box impression casts were transported and scanned into the CAD/CAM system on another site, 8.1% (7/87) reported that the casts were scanned on the site where the patient was assessed, and 1.2% (1/87) provided an invalid response by selecting more than one option (Figure 2e). Just over half (58.2%, (53/91)) of Trusts/HBs reported they occasionally used slipper/plaster casts to capture patients' foot shape when prescribing CAD/CAM insoles, and 3.3% (3/91) did not respond (Figure 2f). With regard to the location of scanning, 96.2% (51/53) of Trusts/HBs confirmed that the plaster/slipper casts would be transported to another site to be scanned into the CAD/CAM system, 1.9% (1/53) scanned the casts on the site where the patient was assessed, and 1.9% (1/53) provided an invalid response by selecting more than one option (Figure 2g).

The majority of Trusts/HBs (86.8% (79/91)) confirmed that they most commonly used foam box impression casts when manufacturing CAD/CAM insoles, 2.2% (2/91) did not respond, 1.1% (1/91) provided a free text answer of “direct scanner”, 1.1% (1/91) selected direct 3D scan using a handheld scanner, 1.1% (1/91) chose direct 3D scan using a flatbed scanner, 1.1% (1/91) chose slipper cast/plaster cast, and 6.6% (6/91) selected two options (Figure 2h). For the 2021/22 financial year, the minimum total number of CAD/CAM insoles produced using foam box impression casts was 36,316, with 3252 produced with direct scanning, and 1288 produced using slipper casts.

With regards to rectifying/modeling the CAD/CAM insoles, 73.6% (67/91) were conducted by a technician, 8.8% (8/91) confirmed the modeling was completed by the orthotist who assessed the patient, 1.1% (1/91) used a clinical assistant, 1.1% (1/91) reported two options, 12.1% (11/91) did not know due to an external manufacturer being responsible for the process, 1.1% (1/91) entered a free text answer of “podiatrist,” and 2.2% (2/91) did not respond (Figure 2i). Therefore the summation of responses from this section of the FOI request shows that the most common workflow for CAD/CAM insoles in UK NHS orthotic services is a hybrid workflow, comprising elements of traditional manufacture and digital techniques (Figure 5).

### 3.4 Barriers and facilitators for CAD/CAM

**FIGURE 3:**
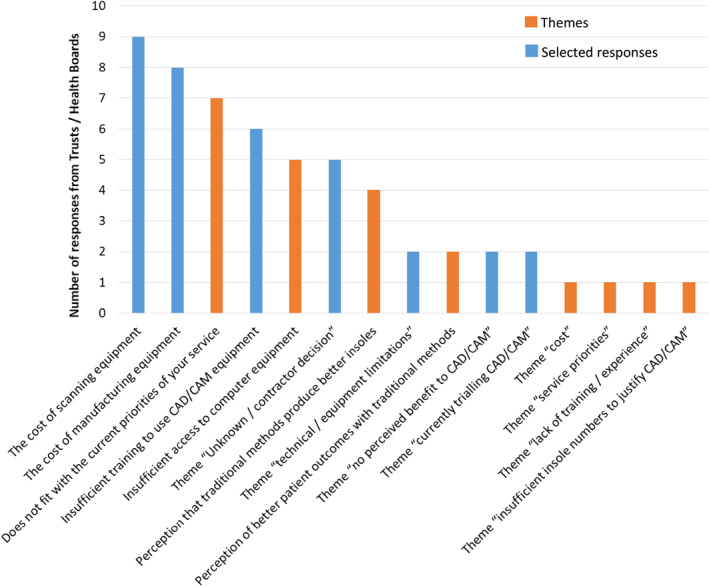
What are the barriers for using computer aided manufacture for custom insoles in your orthotic service?

In order to understand the barriers and facilitators that services experience when considering the use of CAD/CAM for insole manufacture, we asked those respondents who did not use CAD/CAM (25 of the 129 Trusts/HBs) to provide reason(s) for not using CAD/CAM. Multiple responses were permitted, and where a free text response was provided (48% (12/25)), these responses were collated into themes (Table 3). Responses were received from 88% (22/25) Trusts/HBs, with the most common barriers being the cost of scanning equipment (40.9% (9/22), and the cost of manufacturing equipment (36.4% (8/22)), with all selected and thematic responses shown in Figure 3.

**TABLE 3:** Thematic breakdown of free text responses describing barriers to CAD/CAM from individual Trusts and Health Boards.

Trust/Health board response	Cost	Lack of training/experience	Service priorities	Technical/equipment limitations	No perceived benefit to CAD/CAM	Insufficient insole numbers to justify CAD/CAM	Unknown/contractor decision	Currently trialling CAD/CAM
“We have equipment to consider using CAD/CAM but due to this not being top priority, lack of experience, cost for technical support and time, this has been put on hold”	•	•	•					
“They are currently trialling this”								•
“…The numbers of specialist custom made foot orthoses required are lower and thus the cost benefits and time saving of foot only CAD CAM systems are less”						•		
“Our current supplier does not use scanning”							•	
“Unable to answer as this would be a contractor decision”							•	
“This is being considered however the current computer set up may provide difficulties in supporting scanning devices"				•				
“Not offered by the company”							•	
“A good service is provided via the methods currently use”					•			
“We use a company who are just testing the technology”							•	•
“Sharing of information electronically with third parties, not a limiting factor, but one to be considered”				•				
“Poor results with previous CAD systems”					•			
“Unsure, as external contractor”							•	
Totals	1	1	1	2	2	1	5	2

**FIGURE 4:**
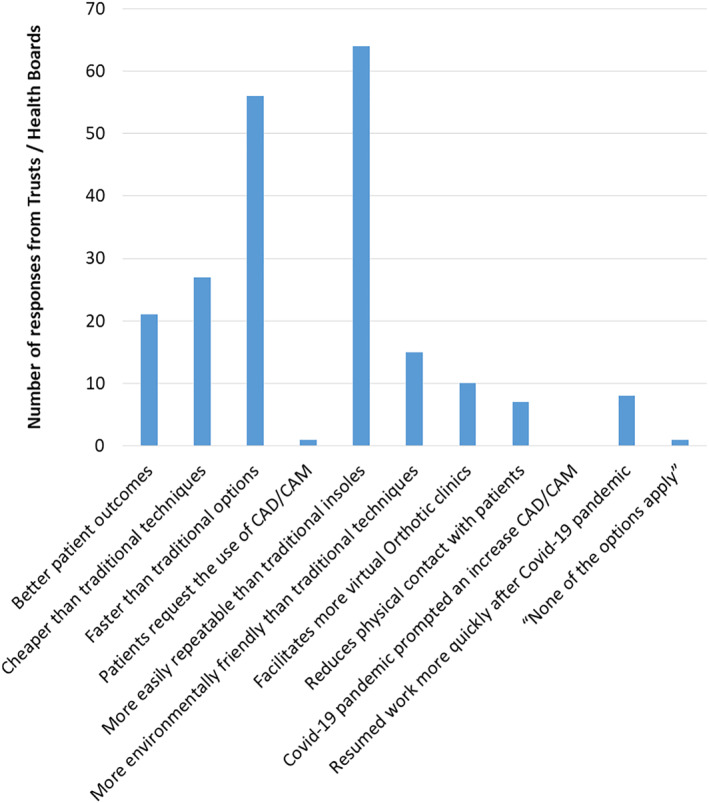
In your orthotic service, what are the reasons for using CAD/CAM insoles?

Those services who did use CAD/CAM (*n* = 91) were asked to select any relevant options from a list of facilitators. Responses were received from 86.8% (79/91) Trusts/HBs with one respondent stating that none of the options applied. The most popular reasons for using CAD/CAM were the perception that CAD/CAM insoles are easily repeatable than traditional insoles (81.0% (64/79)) and CAD/CAM is faster than traditional options (70.9% (56/79)) (Figure 4).

## 4 DISCUSSION

**FIGURE 5:**
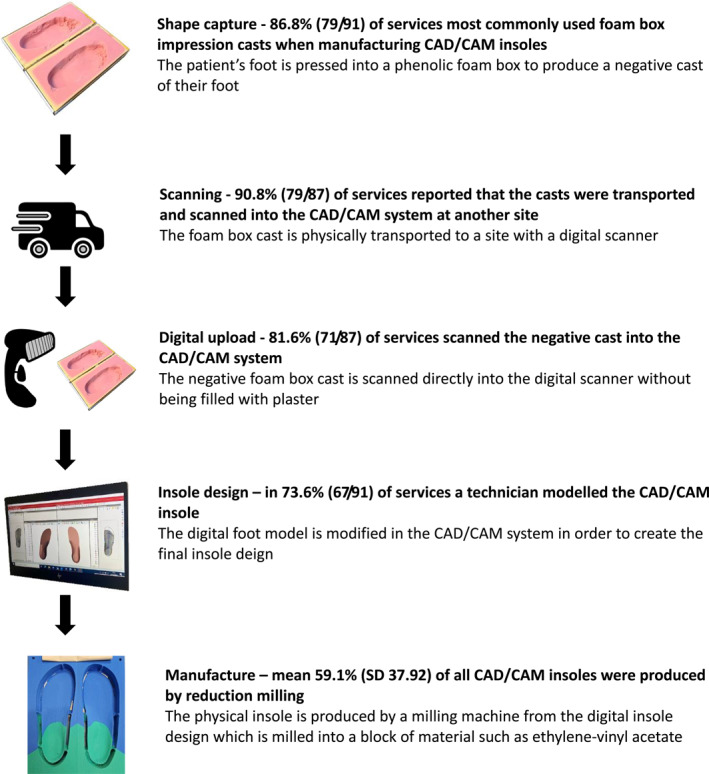
The most common workflow for CAD/CAM insole production in UK orthotic services.

This study was undertaken to gain an understanding of the current practices associated with the provision of CAD/CAM insoles in UK orthotic services. The majority of NHS Trusts/HBs confirmed they did use CAD/CAM as part of their bespoke insole manufacture process, which is in keeping with the anticipated increase in CAD/CAM technology reported in the literature [5, 6]. However, the workflow predominantly used by UK orthotic services (Figure 5) constitutes a hybrid‐digital process rather than a fully‐digital process, whereby some steps associated with traditional manufacture remain. This would potentially reduce some of the reported benefits associated with CAD/CAM such as waste production and speed of manufacture.

Past research has shown that hybrid digital processes, equivalent to the most common process used in the UK as described in this paper, produce greater waste products and pollution, and score less favorably in terms of sustainability than fully digital processes [17]. Furthermore, services using plaster casts and slipper casts within their CAD/CAM insole workflow, as well as those choosing to fill foam box casts with plaster prior to digital upload, further decrease sustainability of the insole production [17]. Although some studies have identified the potential for recycling of both gypsum and plaster of Paris, these techniques are not currently part of routine medical or industrial processes [18, 19, 20]. The production of such avoidable waste products should be strongly considered by orthotic services wishing to improve their environmental impact in terms of carbon emissions, and for those services in the UK to meet NHS net‐zero goals [21, 22]. Future studies comparing patient outcomes using hybrid digital and fully digital workflows may help to better inform orthotic services about the clinical impact of these different methods, in order to support the case for best practice in terms of clinical goals alongside sustainability policies. Despite the increasing development of additive manufacture techniques in the orthotic industry in recent years [23, 24, 25], this study found that additive manufacture was the least used manufacture method for insoles in UK NHS orthotic services. As this is still a relatively new manufacturing technique it is possible that health services have not yet had the opportunity to fully explore the position of additive manufacture in treatment pathways, and future studies will be required to demonstrate any change in practices in the years to come.

With the majority of orthotic services physically transporting casts externally prior to digital upload into the CAD/CAM system, consideration should be given not only to the manufacture delay incurred by this step, but also to the possible carbon emissions associated with transportation [26, 27, 28]. It would therefore be advantageous to compare this with alternative fully‐digital workflows which remove the need for transportation, such as direct scanning, to assess if these processes produce equivalent outcomes in terms of patient treatment, in order to establish best practice for CAD/CAM insole production. The size of the medical foot orthotic industry is expected to increase globally with a compound annual growth rate of 4.6%, in excess of $3.9 billion by 2030 [17], establishing the optimal CAD/CAM processes in terms of clinical effectiveness and sustainability should be a research priority for the orthotic profession.

The greatest barrier to the use of CAD/CAM for insole production was related to equipment costs (Figure 3), which was in keeping with the barriers identified in previous reports [4, 10, 12]. Despite this, cost was also identified as a facilitator to the use of CAD/CAM, with 34.2% (27/79) of Trusts/HBs reporting that using CAD/CAM for insole production was cheaper than traditional techniques. It is possible that those who had not yet introduced CAD/CAM into their service model were limited by start‐up costs associated with the integration of equipment and training of the workforce which has historically incurred high in‐house costs [5, 10]. However, the contradiction observed in this study between the perceptions of cost both as a barrier and a facilitator suggests that services may well be basing their cost concerns on a legacy of historical CAD/CAM prices, which have reduced significantly in recent years such that CAD/CAM technologies are now being recommended as the lowest cost option for low income countries [29].

Lack of experience and training related to the CAD/CAM process were highlighted as a barrier by six of 25 Trusts/HBs, accounting for 12.7% of the total reasons given by services for not using CAD/CAM for insole production. This lack of skills in the UK orthotic profession was also highlighted in the recent prosthetic and orthotic workforce survey, in which only 30% of orthotists reported that they had CAD/CAM skills [30]. Although within the current study, services who did use CAD/CAM were not asked to identify any barriers to their use of CAD/CAM processes, it is possible that the lack of clinical skills relating to scanning and digital modeling could partly explain why the current workflow in the UK favors a hybrid‐digital model, whereby the scanning and modeling are undertaken at a central fabrication centre rather than by the orthotist in charge of the patients' care. In 2020, research on fully‐digital workflows was more than three times greater than that on hybrid‐digital workflows [17]. Workforce reviews have identified that improving clinicians CAD/CAM modeling skills could be a strategic advantage for the profession [11], and over 70% of orthotists believe that CAD/CAM skills will be required by the profession in the future [30]. As such, additional training and support will be necessary before UK orthotic services can transition to fully digital workflows.

In spite of the published benefits that CAD/CAM could offer during the time of the Covid‐19 pandemic [4, 7, 9], none of the Trusts/HBs in our study reported increased use of CAD/CAM for insole production as a result of this, although eight Trusts/HBs were able to resume services more rapidly following the pandemic when they used CAD/CAM. These findings highlight that CAD/CAM processes were already established within these Trusts/HBs at the time of the pandemic, with a median of 10 years duration of use and as such the benefits were already established. Despite the access to digital modeling systems, and increased speed of CAD/CAM modeling, this study found that the majority of services still use a technician to model the digital scans. Although it has previously been suggested that optimal orthotic design would be achieved if the modeling was undertaken by the clinician responsible for the patients' care [7], however it is unknown how this aspect of the manufacturing process impacts on the clinical effectiveness of the final insole. Further research is required to understand how a hybrid‐digital workflow compares with a fully‐digital workflow in terms of patient outcomes, overall costs, and long‐term sustainability.

### 4.1 Limitations

Some Trusts/HBs were unable to provide details for certain aspects of the insole manufacture process due to these being carried out by an external company. Only those Trusts/HBs who did not use CAD/CAM were asked to explain the barriers. This limited the ability to identify the barriers faced by those services who do use CAD/CAM processes within a fully‐digital workflow.

## 5 CONCLUSION

This study has identified considerable variations in processes currently associated with CAD/CAM insole production in UK orthotic services. A hybrid‐digital workflow was found to be the most commonly used in the UK, which has been associated with increased waste products and greater transportation costs compared with a fully‐digital workflow. Those services who are not currently utilising CAD/CAM in their insole workflow predominantly highlighted equipment costs and staff training as the main barriers. Services should consider engaging their staff in CAD/CAM training which has previously been identified as a priority for the future of the profession.

## AUTHOR CONTRIBUTIONS

**Laura Barr**: Funding acquisition; conceptualization; methodology; data curation; formal analysis; writing—original draft; writing—review and editing. **Jim** **Richards**: Methodology; formal analysis; supervision; writing—review and editing. **Graham J. Chapman**: Methodology; formal analysis; supervision; writing—review and editing.

## CONFLICT OF INTEREST STATEMENT

The authors declare no conflicts of interest.

## ETHICS STATEMENT

Approval for the study was received from the Health Ethics Review Panel at the University of Central Lancashire (HEALTH 0365 Phase 2).

## Supporting information

Supporting Information S1

Supporting Information S2

## Data Availability

The data that support the findings of this study are available from the corresponding author upon reasonable request.
