# Supplementary material for: The use of computer‐aided design and manufacture for foot orthoses: A cross‐sectional study of orthotic services in the UK
Source: J Foot Ankle Res. 2025 Feb 5;18(1):e70031. doi: 10.1002/jfa2.70031 (PMC11798744; doi:10.1002/jfa2.70031)
Supplement: Supplementary file 1 — Supporting Information S1 [file JFA2-18-e70031-s002.doc]

**Supplementary File 1.** Freedom of Information Request sent to Trusts and Health Boards

This form has 7 pages. Unless you are prompted to skip a question or to stop answering, please ensure you answer **all** questions before returning the form.

**Section 1**

1. Does your Trust/Health Board have an orthotics department?

☐ Yes

☐ No

**If the answer is “Yes” please answer section 2. If the answer is “No” no further information is required**

**--------------------------------------------------------------------------------------------------------------------------------------**

**Section 2**

- 1. Which of the following best describe your Orthotic Service?
     (select **all that apply** option by entering “X” in the left-hand column)

|  | NHS In-house service (This means the orthotists are directly employed by your Trust/Health Board) |
| --- | --- |
|  | NHS Contracted service (This means an external contractor employs the orthotists) |

- 1. Does your Orthotic Service provide bespoke insoles to patients?
     (select **only one** option by entering “X” in the left-hand column)

|  | Yes *(continue to question 2.3)* |
| --- | --- |
|  | No ***(end of questionnaire)*** |

- 1. How many bespoke insole orders did your Orthotic service place in the 2021/22 financial year?
     (In this context we assume that a “bespoke insole order” is likely to be either a pair of insoles for one patient, or a single insole for one patient)

|  |
| --- |

- 1. Does your Orthotic Service ever provide bespoke insoles which have been **manufactured** using computer-aided processes, such as addition manufacture/3D printing, or reduction manufacture/milling insoles from a digital scan?
     (Select **only one** option by entering “X” in the left-hand column)

|  | Yes *(skip to question 2.6)* |
| --- | --- |
|  | No *(continue to question 2.5)* |

- 1. What are the barriers for using computer aided manufacture for custom insoles in your Orthotic service? (Select **all that apply** by entering “X” in the left-hand column).

|  | The cost of scanning equipment | |
| --- | --- | --- |
|  | The cost of manufacturing equipment (millers, 3D printers, etc.) | |
|  | Insufficient access to computer equipment to support CAD/CAM systems | |
|  | Computer aided manufacture does not fit with the current priorities of your service | |
|  | Insufficient training to use CAD/CAM equipment | |
|  | Perception that traditional methods produce better insoles | |
|  | Perception of better patient outcomes with traditional methods | |
|  | Other. Please provide a reason in the right-hand column | Free-text reason: |

**If you have completed question 2.5, this is now the end of the form.

If you were not asked to complete question 2.5 you should continue to the next page.**

- 1. Which methods are used to **manufacture** bespoke insoles in your Orthotic service? (Select **all that apply** by entering “X” in the left-hand column)

|  | **In-house Traditional**. You have staff on site in your service who use heat moulding / draping techniques to produce the insole |
| --- | --- |
|  | **In-house Computer Aided Manufacture using Reduction Manufacture**. You have milling equipment on site in your service and mill insoles from a block of material |
|  | **In-house Computer Aided Manufacture using Additive Manufacture**. You have a “3Dprinter” on site in your service and manufacture insoles using additive processes |
|  | **Outsourced Traditional.** Your casts or models are sent to an external technical company who use heat moulding / draping techniques to produce the insole |
|  | **Outsourced Computer Aided Manufacture using Reduction Manufacture.** Your casts, models or scans are sent to an external technical company who mill insoles from a block of material |
|  | **Outsourced Computer Aided Manufacture using Additive Manufacture.** Your casts, models or scans are sent to an external technical company who manufacture the insoles using an additive process / “3D printer” |
|  | **Do not know -** only select this option if your insoles are usually manufactured externally and you do not have knowledge of the external processes |

**Questions continue on next page**

**The definitions for the terms used in these questions, are explained on page 3.**

- 1. In your Orthotic service, what **percentage** of insoles were made using
     **In-house Traditional Manufacture** in the 2021/22 financial year?

| % |
| --- |

- 1. In your Orthotic service, what **percentage** of insoles were made using
     **In-house Computer Aided Manufacture with Reduction Manufacture** in the 2021/22 financial year?

| % |
| --- |

- 1. In your Orthotic service, what **percentage** of insoles were made **using In-house Computer Aided Manufacture with Additive Manufacture (3D printed)** in the 2021/22 financial year?

| % |
| --- |

- 1. In your Orthotic service, what **percentage** of insoles were made using **Outsourced Traditional Manufacture** in the 2021/22 financial year?

| % |
| --- |

- 1. In your Orthotic service, what **percentage** of insoles were made using **Outsourced Computer Aided Manufacture with Reduction Manufacture** in the 2021/22 financial year?

| % |
| --- |

- 1. In your Orthotic service, what **percentage** of insoles were made using **Outsourced Computer Aided Manufacture with Additive Manufacture (3D printed)** in the 2021/22 financial year?

| % |
| --- |

**Questions continue on next page**

**Section 3**

The following questions relate **only** to the insoles produced by computer aided design and manufacture (CAD/CAM). These may be manufactured in-house or externally.
If your service and/or insole manufacturer do not use this method, you do not need to answer any further questions.

- 1. How long has your Orthotic service provided bespoke insoles to patients, which were produced using computer aided manufacture processes?

|  |
| --- |

- 1. Does your Orthotic service ever use foam box impression casts to capture the shape of the patient’s foot, when prescribing CAD/CAM insoles? (Select **only one** option by entering “X” in the left-hand column)

|  | Yes *(continue to question 3.3)* |
| --- | --- |
|  | No *(skip to question 3.4)* |

- 1. Is the negative foam box impression cast **usually** scanned into the CAD/CAM system, or is it filled with plaster first and then the positive model scanned? (Select **only one** option by entering “X” in the left-hand column)

|  | The negative foam box is usually scanned |
| --- | --- |
|  | The foam box is usually filled with plaster and the positive cast is then scanned |
|  | Do not know – only select this option if your insoles are usually manufactured externally and you do not have knowledge of the external processes |

- 1. Are the foam box impression casts usually transported to another site to be scanned into the CAD/CAM system? (Select **only one** option by entering “X” in the left-hand column)

|  | Yes - they are usually sent to another hospital or external manufacturer to be filled with plaster and/or scanned |
| --- | --- |
|  | No - they are usually scanned on the same site that the patient attended for their appointment |

- 1. Does your Orthotic service ever use slipper casts / plaster casts to capture the shape of the patient’s foot, when prescribing CAD/CAM insoles?
     (Select **only one** option by entering “X” in the left-hand column)

|  | Yes *(continue to question 3.4)* |
| --- | --- |
|  | No *(skip to question 3.5)* |

- 1. Are the slipper casts / plaster casts usually transported to another site to be filled with plaster and scanned into the CAD/CAM system?
     (Select **only one** option by entering “X” in the left-hand column)

|  | Yes – they are usually sent to another hospital or external manufacturer to be filled with plaster and / or scanned |
| --- | --- |
|  | No – they are usually filled with plaster and scanned on the same site that the patient attended for their appointment |

- 1. In your Orthotic service, which is the **most common method** used to capture the shape of the patient’s foot, when prescribing CAD/CAM insoles
     (Select **only one** option by entering “X” in the left-hand column)

|  | Direct 3D scan using a flat-bed scanner | |
| --- | --- | --- |
|  | Direct 3D scan using a handheld scanner | |
|  | Foam box impression cast | |
|  | Slipper cast / plaster cast | |
|  | Measurements only (using tracings or tape measures etc.) | |
|  | Other. Please specify in the right-hand column | Free-text: |

**Questions continue on next page**

- 1. Who is **usually** responsible for performing the modelling/rectification of the CAD/CAM insoles that your Orthotic service provide?
     (Select **only one** option by entering “X” in the left-hand column)

|  | The orthotist who assessed the patient | |
| --- | --- | --- |
|  | Another orthotist who did not assess the patient | |
|  | A clinical assistant | |
|  | A technician | |
|  | Do not know – only select this option if your insoles are usually manufactured externally and you do not have knowledge of the external processes | |
|  | Other. Please specify in the right-hand column | Free-text: |

- 1. In your Orthotic service, what are the reasons for using CAD/CAM insoles? (Select **all options that apply** by entering “X” in the left-hand column)

|  | Perception that CAD/CAM insoles produce better patient outcomes |
| --- | --- |
|  | CAD/CAD production is cheaper for us than traditional techniques |
|  | CAD/CAM insole production is faster than the traditional options |
|  | The production of CAD/CAM insoles is more environmentally friendly than traditional techniques |
|  | Patients request the use of CAD/CAM |
|  | CAD/CAM insoles are more easily repeatable than traditional insoles |
|  | Producing insoles with CAD/CAM facilitates us in running more virtual Orthotic clinics |
|  | Producing insoles with CAD/CAM allows us to reduce physical contact with patients |
|  | The Covid-19 pandemic prompted us to increase the use of CAD/CAM insole production |
|  | Producing insoles with CAD/CAM allowed our Orthotic service to resume work more quickly following the onset of the Covid-19 pandemic |

**END OF QUESTIONS**
