# Supplementary material for: The use of computer‐aided design and manufacture for foot orthoses: A cross‐sectional study of orthotic services in the UK
Source: J Foot Ankle Res. 2025 Feb 5;18(1):e70031. doi: 10.1002/jfa2.70031 (PMC11798744; doi:10.1002/jfa2.70031)
Supplement: Supplementary file 2 — Supporting Information S2 [file JFA2-18-e70031-s001.doc]

**Supplementary File 2.** Response rates for individual questions

Question 2.1: Which of the following best describe your Orthotic Service?

| **Service type % (number of replies)** |
| --- |
| NHS In-house service 31.3% (41/131) |
| NHS Contracted service 61.8% (81/131) |
| Both 6.9% (9/131) |
| No response 0% (0/131) |

Question 2.2: Does your Orthotic Service provide bespoke insoles to patients?

| **Response % (number of replies)** |
| --- |
| Yes 93.1% (122/131) |
| No 1.5% (2/131) |
| No response 5.3% (7/131) |

Question 2.3: How many bespoke insole orders did your Orthotic service place in the 2021/22 financial year?*

| **Response % (number of replies)** |
| --- |
| Response received 80.6% (104/129)# |
| No response 19.4% (25/129) |

*Those who responded “no” to question 2.2 were not required to answer the remainder of the questions, therefore the total number of possible respondents is 129
#Of these respondents 15/129 stated that their response was an estimate or a range rather than a known value

Question 2.4: Does your Orthotic Service ever provide bespoke insoles which have been **manufactured** using computer-aided processes, such as addition manufacture/3D printing, or reduction manufacture/milling insoles from a digital scan?

| **Response % (number of replies)** |
| --- |
| Yes 70.5% (91/129) |
| No 19.4% (25/129) |
| No response 10.1% (13/129) |

Question 2.5: What are the barriers for using computer aided manufacture for custom insoles in your Orthotic service?*

| **Response % (number of replies)** |
| --- |
| Response received 92% (23/25) |
| No response 8% (2/25) |

*Only those who responded “No” to question 2.4 were required to answer. Therefore the total number of possible respondents is 25.

Question 2.6: Which methods are used to **manufacture** bespoke insoles in your Orthotic service?*

| **Response % (number of replies)** |
| --- |
| Response received 94.5% (86/91) |
| No response 5.5% (5/91) |

* Only those who responded “Yes” to question 2.4 were required to answer the remaining questions. Therefore the total number of possible respondents is 91.

**Questions 2.7 to 2.12: In your Orthotic service, what percentage of insoles were made using**

| 2.7 In-house Traditional Manufacture. **Response % (number of replies)** | 2.8. In-house Computer Aided Manufacture with Reduction Manufacture. **Response % (number of replies)** | 2.9. In-house Computer Aided Manufacture with Additive Manufacture. **Response % (number of replies)** | 2.10 Outsourced Traditional Manufacture. **Response % (number of replies)** | 2.11. Outsourced Computer Aided Manufacture with Reduction Manufacture. **Response % (number of replies)** | 2.12. Outsourced Computer Aided Manufacture with Additive Manufacture. **Response % (number of replies)** |
| --- | --- | --- | --- | --- | --- |
| Response received 86.8% (79/91) | Response received 86.8% (79/91) | Response received 86.8% (79/91) | Response received 79.1% (72/91) | Response received 79.1% (72/91) | Response received 84.6% (77/91) |
| No response 13.2% (12/91) | No response 13.2% (12/91) | No response 13.2% (12/91) | No response 20.9% (19/91) | No response 20.9% (19/91) | No response 15.4% (14/91) |

Question 3.1: How long has your Orthotic service provided bespoke insoles to patients, which were produced using computer aided manufacture processes?

| **Response % (number of replies)** |
| --- |
| Response received 85.7% (78/91) |
| No response 14.2% (13/91) |

Question 3.2: Does your Orthotic service ever use foam box impression casts to capture the shape of the patient’s foot, when prescribing CAD/CAM insoles?

| **Response % (number of replies)** |
| --- |
| Yes 95.6% (87/91) |
| No 1.1% (1/91) |
| No response 3.3% (3/91) |

Question 3.3: Is the negative foam box impression cast **usually** scanned into the CAD/CAM system, or is it filled with plaster first and then the positive model scanned?*

| **Response % (number of replies)** |
| --- |
| Response received 100.0% (87/87) |
| No response 0.0% (0/87) |

* Only those who responded “Yes” to question 3.2 were required to answer this question. Therefore the total number of possible respondents is 87.

Question 3.4: Are the foam box impression casts usually transported to another site to be scanned into the CAD/CAM system?

| **Response % (number of replies)** |
| --- |
| Response received 100.0% (87/87) |
| No response 0.0% (0/87) |

* Only those who responded “Yes” to question 3.2 were required to answer this question. Therefore the total number of possible respondents is 87.

Question 3.5: Does your Orthotic service ever use slipper casts / plaster casts to capture the shape of the patient’s foot, when prescribing CAD/CAM insoles?

| **Response % (number of replies)** |
| --- |
| Yes 58.2% (53/91) |
| No 38.5% (35/91) |
| No response 3.3% (3/91) |

Question 3.6: Are the slipper casts / plaster casts usually transported to another site to be filled with plaster and scanned into the CAD/CAM system?

| **Response % (number of replies)** |
| --- |
| Response received 100.0% (53/53) |
| No response 0.0% (0/53) |

* Only those who responded “Yes” to question 3.5 were required to answer this question. Therefore the total number of possible respondents is 53.

Question 3.7: In your Orthotic service, which is the **most common method** used to capture the shape of the patient’s foot, when prescribing CAD/CAM insoles

| **Response % (number of replies)** |
| --- |
| Response received 97.8% (89/91) |
| No response 2.2% (2/91) |

Question 3.8: Who is **usually** responsible for performing the modelling/rectification of the CAD/CAM insoles that your Orthotic service provide?

| **Response % (number of replies)** |
| --- |
| Response received 97.8% (89/91) |
| No response 2.2% (2/91) |

Question 3.9: In your Orthotic service, what are the reasons for using CAD/CAM insoles?

| **Response % (number of replies)** |
| --- |
| Response received 86.8% (79/91) |
| No response 13.2% (12/91) |
